# Anxiety and depression among adult tobacco users during the COVID-19 restrictions in India

**DOI:** 10.3389/fpsyt.2022.964949

**Published:** 2022-08-23

**Authors:** Shalini Bassi, Gaurang P. Nazar, Nishigandha Joshi, Nitika Sharma, Aishwarya Pandian, Mohan Deepa, Sailesh Mohan, Shivani A. Patel, Mohammed K. Ali, Ann McNeill, Nikhil Tandon, Viswanathan Mohan, Dorairaj Prabhakaran, Monika Arora

**Affiliations:** ^1^Health Promotion Division, Public Health Foundation of India, New Delhi, India; ^2^Health Related Information Dissemination Amongst Youth (HRIDAY), New Delhi, India; ^3^Los Angeles County Department of Public Health, Los Angeles, CA, United States; ^4^Madras Diabetes Research Foundation & Dr. Mohan’s Diabetes Specialities Centre, Chennai, Tamil Nadu, India; ^5^Centre for Chronic Conditions and Injuries (CCCI), Public Health Foundation of India & Centre for Chronic Disease Control, New Delhi, India; ^6^Hubert Department of Global Health, Emory University, Atlanta, GA, United States; ^7^King’s College London, London, United Kingdom; ^8^Department of Endocrinology, All India Institute of Medical Sciences, National Capital Territory of Delhi, New Delhi, India

**Keywords:** mental health, tobacco users, COVID-19, lockdown, anxiety, depression

## Abstract

**Background:**

The world witnessed a highly contagious and deadly disease, COVID-19, toward the end of 2019. India is one of the worst affected countries. We aimed to assess anxiety and depression levels among adult tobacco users and people who recently quit tobacco during COVID-19 lockdown in India.

**Materials and methods:**

The study was conducted across two Indian cities, Delhi and Chennai (July-August, 2020) among adult tobacco users (*n* = 801). Telephonic interviews were conducted using validated mental health tools (Patient Health Questionnaire-PHQ-9 and Generalized Anxiety Disorder-GAD-7) to assess the anxiety and depression levels of the participants. Descriptive analysis and multiple logistic regression were used to study the prevalence and correlates of depression and anxiety.

**Results:**

We found that 20.6% of tobacco users had depression symptoms (3.9% moderate to severe); 20.7% had anxiety symptoms (3.8% moderate to severe). Risk factors associated with depression and anxiety included food, housing, and financial insecurity.

**Conclusion:**

During COVID-19 lockdown, mental health of tobacco users (primarily women) was associated with food, housing and financial insecurity. The Indian Government rightly initiated several health, social and economic measures to shield the most vulnerable from COVID-19, including a ban on the sale of tobacco products. It is also necessary to prioritize universal health coverage, expanded social security net, tobacco cessation and mental health services to such vulnerable populations during pandemic situations.

## Introduction

The novel Coronavirus or SARS CoV-2, which began in late 2019, has infected more than 25 million people worldwide thus far and still increasing (1). Besides the massive human toll and economic burden on the Indian healthcare system, the pandemic also posed a myriad of challenges for the country’s public health system. As the primary preventive strategy, India went under a nationwide lockdown in March 2020 (2). Strict lockdown regulations and, most importantly, the forced social confinement, disrupted the supply chain of essential commodities, affecting the nation’s mental health as a whole (3, 4). Fear, anxiety, depression, and insomnia were a few common mental health issues detected in the population during the lockdown (5, 6). COVID-19 Mental Disorders Collaborators concluded that the pandemic led to a 27⋅6% increase in cases of major depressive disorders and 25⋅6% increase in cases of anxiety disorders, globally (7). There is a complex relationship between COVID-19 and smoking. Tobacco users are at increased risk of adverse outcome like death and severity of COVID-19 infection (8). Considering this, the Government of India issued several advisories to prohibit the use and spitting of tobacco in public places (9). Subsequently, more stringent tobacco control measures were implemented in India such as banning the sale and use of tobacco products in public places to prevent spitting during COVID-19 (10). A cross-sectional study found that nearly half of the users couldn’t easily access tobacco products during this period (11). There are complex and often bidirectional dynamics between substance abuse and mental health problems (12, 13) which are often believed to co-exist (14).

A study conducted following the outbreak of COVID-19, suggested increased mental health problems during the pandemic was associated with increased tobacco use as well (15). However, there is conflicting as well scant scientific literature regarding mental health disorders among tobacco users in India, particularly considering the realities of the pandemic.

While we are trying to cope and adapt to the new normal with all the focus on containing the pandemic, the mental health impact due to the unprecedented situation created during these times remains unaddressed. These challenges pose even a more significant threat, especially in tobacco consumer groups, especially when there is no capacity and networks for provision of psychosocial support during these vulnerable times. The research evidence reveals significant associations were observed between different smoking behavior groups and psychosocial factors (15). Therefore, there is an urgent requirement to understand and address the psychological burden of tobacco users as well. Hence, this cross-sectional study was conducted to assess the prevalence and correlates of anxiety and depression among adult tobacco users during the COVID-19 restrictions in India.

## Materials and methods

### Study design, setting, and participants

A cross-sectional study was conducted in New Delhi and Chennai (India) between July and August 2020. The study was conducted with adult tobacco users (*n* = 801), both males (*n* = 722) and females (*n* = 79). The inclusion criteria being participant’s consent to participate, aged 20 years or above, people who can understand Hindi, English, and Tamil, and be a current tobacco user (any form of tobacco). The participants who were institutionalized, unable to respond the survey, not speak or understand Hindi, English, or Tamil and not willing to provide or record verbal consent were excluded from the study. The participants who used tobacco in any form in the past one month (from the onset of the survey, i.e, July 2020) or have quit tobacco during past three months (from the onset date of the survey) were included in the survey. The study participants were recruited from the pre-existing cohort of the CARRS study (”Centre for Cardiometabolic Risk Reduction in South Asia-CARRS), a model surveillance system for cardio-metabolic diseases (16). Considering the large target population (over 1 million), assuming a 5% margin of error, with a 95% confidence level, we estimated a minimum sample size of ∼800 for our study.

### Data collection

The study data were collected through telephonic interviews, administered by a trained research team, using a standardized protocol. The telephonic interview technique was adopted for data collection to counter the spread of COVID-19 and protect all individuals associated. The questionnaires were administered in English, Hindi, or Tamil, based on the participants’ preferences. Team of researchers were skilled to conduct interviews in the respective languages. Those eligible to participate were then asked for informed consent. The verbal consent was audio-recorded following the Indian Council of Medical Research’s revised guidelines for obtaining consent for biomedical and health research during the COVID-19 pandemic (17). This method was approved by the Ethics Committee. Prior ethics approval for the research involving human subjects was obtained from the Centre for Chronic Disease Control’s Institutional Ethics Committee (Reference # CCDC_IEC_04_2018).

### Study instruments and measures

A validated mental health tool, i.e., Patient Health Questionnaire-9 (PHQ-9), was used in our study to assess the symptoms of depression and anxiety among tobacco users (18, 19). Participants assigned each indicator (e.g., little interest or pleasure in doing things) a value based on the frequency of symptoms they experienced over the preceding two weeks, on a 4-point scale – 0 (not at all), 1 (several days), 2 (more than half the days) and 3 (nearly every day). The total score ranged from zero to 27. The participants were categorized for the severity of depression based on cumulative scores. A score of 4 or lower was dismissed for signs of depression, 5 to 9 fell into mild, 10 to 14 reflected moderate depression, 15 to 19 was moderately severe depression, and anything beyond 20 was severe depression (18). These scores were further re-coded for analysis; scores 4 or lower were coded as 0 or “having no symptoms of depression,” and scores ≥ 5 were coded as 1 or “with depression symptoms” (20).

Likewise, Generalized Anxiety Disorder-7 (GAD-7) was used to assess self-reported symptoms of anxiety (21). The study participants ranked each item (e.g., not being able to stop or control worrying) based on the recurrence of symptoms in the previous two weeks on a 4-point scale – 0 (not at all), 1 (several days), 2 (more than half the days) and 3 (nearly every day). The aggregate score ranged from 0 to 21. Scores of 4 or lower reflected no anxiety, 5 to 9 represented mild anxiety, 10 to 14 indicated moderate anxiety, and 15 to 21 indicated severe anxiety (22). During analysis, scores 0 to 4 were coded as 0 or “having no symptoms of anxiety,” and scores ≥ 5 were coded as 1 or “with symptoms of anxiety.”

Additional demographic information, namely age, sex, education level, employment status, were gathered. Particulars about the participants’ pre-existing comorbidities (e.g., diabetes, hypertension, stroke, cancer) were obtained from the CARRS database. Additional information on variable definitions is presented in Supplementary Table 1.

### Statistical analysis

Chi-square test and Fisher’s Exact test were used to determine univariate associations between the socio-demographic characteristics and depression, and anxiety. Unpaired (two-sample) *t*-test was used to compare the mean score of depression and anxiety among male and female participants. The threshold for significance was set at *p* < 0.05. The data are displayed as a mean score ± (SD), proportions, and percentages. Multiple logistic regression analysis was used to determine Odds Ratios (OR) and 95% Confidence Intervals (95% CI) for associations between dependent variable depression (coded 1 = depression present, 0 = depression absent) and other predictor variables. Participant’s location, sex, age, education, employment status, and other variables like financial status during the lockdown, food, and housing insecurity were treated as independent variables. Similarly, for the other dependent variable, anxiety (coded, 1 = anxiety present, 0 = anxiety absent), the same independent variables were used in the regression model. All tests were considered significant at the 0.05 level. The independent variables were examined for multicollinearity. The VIF values were less than ten, indicating no evidence of multicollinearity (18). The data were analyzed using STATA 13.0 (StataCorp, LP, Texas) (23).

## Results

### Study participants’ characteristics

In total, 2,505 adult tobacco users from Delhi (*n* = 1365) and Chennai (*n* = 1140) were approached to participate in our study. A total of 801 tobacco users participated in the survey out of whom, 444 (55.4%) were from Delhi while 357 (44.6%) from Chennai. As the survey was conducted telephonically, a disposition table is used to explain the response rates [Supplementary Tables 2, 3]. The gross response rate for the study was 48.4%, the basic response rate was 85.3% and the response rate calculated using the CASRO Estimator (24) was 60.9%. Roughly 90% of the adult tobacco users were males, and 87.9% were in the age group of 25-64 years. The majority (81.2%) were employed, and 11.5% had a bachelor’s degree and above. The study participants’ mean age was 50.5 years, with a range from 25 to 90 years. The majority of tobacco users were smokeless tobacco users (40.5%), followed by cigarette smokers (38.0%) and bidi smokers (24.3%) (Table 1).

**TABLE 1:** Socio-demographic characteristics of the study participants (*n* = 801).

Socio-demographic characteristics	n (%)
**City**	
Delhi	444 (55.4)
Chennai	357 (44.6)
**Sex**	
Males	722 (90.1)
Females	79 (9.9)
**Age (in years)**	
25–44	253 (31.6)
45–64	451 (56.3)
65 and above	97 (12.1)
**Education**	
Professional Degree/Post Graduate	17 (2.1)
Graduate (B.A/B.Sc./B.Com/Diploma)	75 (9.4)
Secondary School/Intermediary	249 (31.0)
High school (class V to IX)	316 (39.5)
Primary School (up to Class IV)	64 (8.0)
No formal education	80 (10.0)
**Employment status**	
Employed	650 (81.2)
Student	88 (11.0)
Housewife	29 (3.6)
Retired	16 (2.0)
Unemployed	18 (2.2)
Tobacco use*	
Cigarette smokers	305 (38.0)
Cigarette smokers who recently quit	15 (1.9)
Bidi smokers (*n* = 798)	195 (24.3)
Bidi smokers who recently quit (*n* = 195)	10 (1.2)
Smokeless tobacco users (*n* = 800)	324 (40.5)
Smokeless tobacco users who recently quit (*n* = 324)	13 (1.6)
Dual Users	30 (3.7)

*For Cigarette smokers (Out of *n* = 801, 305 were cigarette smokers; Out of them 15 participants had recently quit cigarette smoking).

For Bidi smokers (*n* = 798), three observations were missing; Out of them 195 were bidi smokers; of which 10 participants had recently quit bidi smoking.

For Smokeless tobacco users (*n* = 800), one observation was missing; Out of which 324 participants were smokeless tobacco users; of which 13 participants had recently quit smokeless tobacco.

Recently quit tobacco – Participants who had quit tobacco during past three months (from the onset date of survey).

### Prevalence of depressive symptoms among tobacco users

**FIGURE 1:**
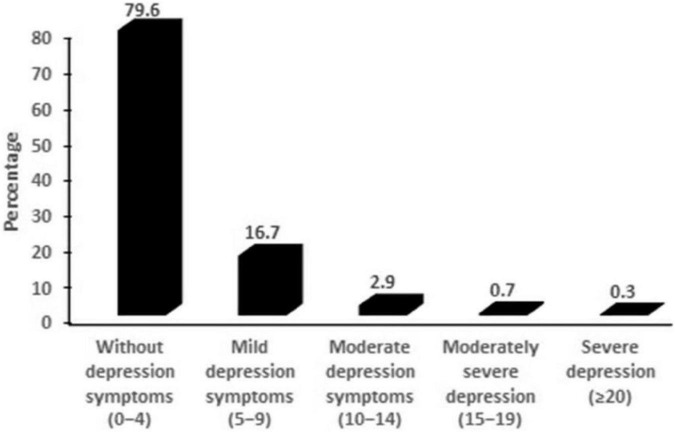
Prevalence of depression symptoms among tabacco users during COVID-19 pandemic.

The mean PHQ-9 score for the study participants was 2.5 ± (3.4). Of the 763 complete responses, 20.6% of tobacco users were found to have depression symptoms (PHQ-9 score > 4). About 16.7% of participants reported mild depression symptoms (PHQ-9 score between 5 and 9), 2.9% experienced moderate depression (PHQ-9 score between 10 and 14), 0.7% had moderately severe depression (PHQ-9 score 15–19), and 0.3% had severe depression (PHQ-9 score 20–27) (Figure 1). The mean depression score for females was 3.2 ± (3.1), which was significantly higher than that for males 2.4 ± (3.4). (*p* = 0.02).

**FIGURE 2:**
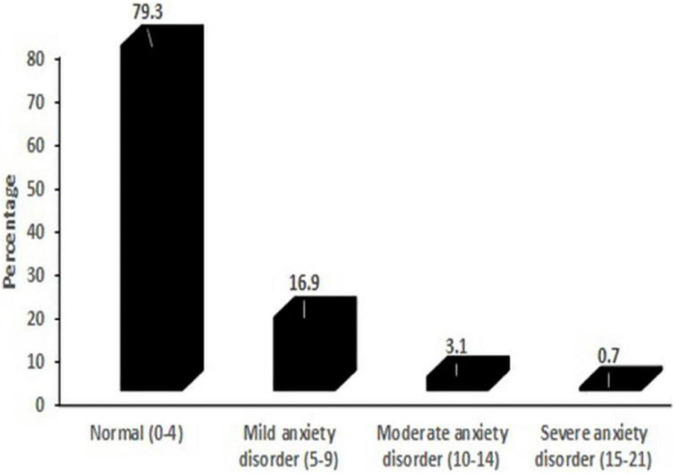
Prevalence of anxiety symptoms among tabacco users during COVID-19 pandemic.

Figure 2 represents the distribution of the tobacco users according to the GAD-7 score. The mean GAD-7 score for the study participants was 2.4 (SD ± 3.2). Of the 774 respondents responding to anxiety questions, 20.7% had a GAD score greater than 4. The prevalence of mild anxiety (GAD score 5–9) was 16.9%, 3.1% had moderate anxiety symptoms (GAD score 10–14), and 0.7% had severe anxiety symptoms (GAD score 15–21). When analyzed along gender lines, the mean anxiety score for females was 3 ± (3.2), significantly higher than the mean scores for males at 2.3 ± (3.20) (*p* = 0.02).

Table 2 shows the univariate associations of depression and anxiety with the socio-demographic as well as other independent variables. The symptoms of anxiety appeared in 27% and depression in 25% of the study participants, who were employed but currently not working, unemployed, or had uncertain employment status. In contrast, among the individuals who were employed and currently working, 17% experienced depression and 16.9% anxiety.

**TABLE 2:** Prevalence of depression and anxiety symptoms among tobacco users.

Variables	Depression (≥ 5) (*N* = 763) n (%)	P value	Anxiety (≥ 5) (*N* = 774) n (%)	P-value
**Sex**				
Males (*N* = 722)	138 (19.1)	0.384	138 (19.1)	0.071
Females (*N* = 79)	18 (22.7)		22 (27.8)	
**Age category (in years)**				
25–44 (*N* = 253)	51 (20.1)	0.896	57 (22.5)	0.552
45–64 (*N* = 451)	86 (19)		85 (18.8)	
65 years and above (*N* = 97)	19 (19.6)		18 (18.6)	
**Education**				
Professional degree/post graduate (*N* = 17)	1 (5.8)	0.851	1 (5.8)	0.523
Graduate (*N* = 75)	15 (20)		11 (14.6)	
Secondary school/Intermediary(*N* = 249)	48 (19.2)		52 (21.9)	
High school (*N* = 316)	64 (20.3)		66 (20.9)	
Primary School(*N* = 64)	12 (18.8)		12 (18.8)	
Illiterate (*N* = 80)	16(20)		18 (22.5)	
**Employment status**				
Employed and currently working (N = 551)	94 (17)	**0.009**	93 (16.9)	**0.001**
Employed but currently not working/unemployed/uncertain employment (*N* = 248)	62 (25)		67 (27)	
**Financial status**				
Doing alright (*N* = 188)	18 (9.6)	**< 0.001**	15 (8)	**< 0.001**
Just about getting by/finding it quite difficult (*N* = 568)	129 (22.7)		140 (24.6)	
**Lockdown status**				
Complete or partial lockdown (*N* = 481)	89 (18.5)	0.609	84 (17.5)	0.706
No lockdown (*N* = 94)	19 (20.2)		18 (19.1)	
**Food Security (ability to buy food during COVID-19 lockdown)**				
Often (*N* = 598)	63 (10.5)	**< 0.001**	93 (16)	**< 0.001**
Sometimes/never (*N* = 192)	44 (23)		66 (34.3)	
**House security (worried about paying rent or house loan)**				
No (*N* = 501)	71 (14.2)	**< 0.001**	61 (12.1)	**< 0.001**
Sometimes (*N* = 83)	20 (24)		20 (24)	
Yes (*N* = 213)	65 (30.5)		79 (37)	
**Worried about getting evicted from house**				
No (*N* = 646)	94 (14.6)	**< 0.001**	86 (13.3)	**< 0.001**
Sometimes (*N* = 60)	22 (36.6)		28 (46.6)	
Yes (*N* = 91)	39 (42.8)		46 (50.5)	
**Cigarette use**				
Yes (*N* = 305)	55 (18)	0.365	57 (18.7)	0.422
No (*N* = 496)	101 (20.4)		103 (20.8)	
**Bidi use**				
Yes (*N* = 195)	32 (16.4)	0.270	30 (15.4)	0.091
No (*N* = 603)	122 (20.2)		128 (21.2)	
**Smokeless tobacco use**				
Yes (*N* = 324)	67 (20.7)	0.558	71 (22)	0.313
No (*N* = 476)	89 (18.7)		89 (18.7)	

*Chi square test and Fisher’s Exact Test (25, 26); For depression (*n* = 763)- 38 Observations were missing; For Anxiety (*n* = 774) – 27 Observations were missing. The bold values are significant values.

Individuals experiencing poor financial status displayed a marked distinction in depression (22.7%) and anxiety symptoms (24.6%) when compared to those who claimed to be financially secure and stable during the pandemic-depression (9.6%) and anxiety (8%). Tobacco users who reported the ability to buy food sometimes or never during COVID-19 lockdown had significantly higher rates of depression (23%) and anxiety symptoms (34.3%) as opposed to (10.5%) and (16%) in people who could often buy food during the pandemic. Of the individuals who were worried about paying house rent or loans, 30.5% claimed to have depression, and 37% reported anxiety symptoms, starkly distinguished from individuals who were not worried had depression (14.2%) and anxiety (12.1%) respectively. The participants who worried about being evicted from homes were significantly more depressed (42.8%) as compared to those who were not worried (14.6%) and reported anxiety symptoms in 50.5% of participants as compared to those who were not worried (13.3%). We observed that the prevalence of depression and anxiety symptoms did not change with the presence of lockdown restrictions or by type of containment zones (*P* > 0.05).

We observed that the presence of depression and anxiety symptoms did not change with the type of tobacco use and did not differ significantly between single and dual/multiple tobacco product users. The people who had recently quit tobacco did not display marked distinction in depression and anxiety symptoms than current tobacco users.

Table 3 represents findings from the logistic regression analysis assessing predictors of depression and anxiety in adult tobacco users during COVID-19. In females, the odds of anxiety were twice that in males (OR = 2, 95% CI 1.0–3.8). The risk of depression was 1.8 times and anxiety 2.2 times among participants who were financially just about getting by or finding it quite difficult as compared to those who were financially doing all right (OR = 1.8, 95% CI 1.0 −3.1 and OR = 2.2, 95% CI 1.2-4.0, respectively). The risk of being depressed was 2.3 times and being anxious, 1.9 times higher in people who could buy food only sometimes or never than in the people who could buy food often during the lockdown (OR 2.3, 95% CI 1.4 – 3.7 and OR 1.9, 95% CI 1.1 – 3.0), respectively.

**TABLE 3:** Predictors of depression and anxiety in adult tobacco users during COVID-19*.

Covariates	Depression (*n* = 715) OR (95% CI)	Anxiety (*n* = 725) OR (95% CI)
**City**		
Chennai	(Ref)	(Ref)
Delhi	1.3 (0.8 – 2.0)	1.2 (0.7 – 1.8)
**Sex**		
Male	(Ref)	(Ref)
Female	1.3 (0.6 – 2.6)	**2 (1.0 – 3.8)**
**Age Category**		
25–44	(Ref)	(Ref)
45–64	1.0 (0.7 – 1.6)	0.8 (0.6 –1.3)
65 years and above	1.0 (0.5 – 2.1)	1.0 (0.5 – 2.1)
**Education**		
Professional degree/post graduate	(Ref)	(Ref)
Graduate	2.6 (0.3 – 21.8)	1.4 (0.2 –12.4)
Secondary school/Intermediary	1.9 (0.2 – 15.5)	1.7 (0.2 – 13.8)
High school	1.8 (0.2 –14.3)	1.3 (0.1 – 10.9)
Primary school	1.8 (0.2 – 16.1)	1.2 (0.1 – 11.3)
Illiterate	1.4 (0.2 – 12.6)	0.9 (0.1 – 8.0)
**Employment status**		
Employed and currently working	(Ref)	(Ref)
Employed but currently not working/unemployed/uncertain employment	1.0 (0.7 – 1.6)	1.2 (0.7 – 1.8)
**Financial status**		
Doing alright	(Ref)	(Ref)
Just about getting by/finding it quite difficult	**1.8 (1.0 –3.1)**	**2.2 (1.2 – 4.0)**
**Food Security (ability to buy food during COVID-19 lockdown)**		
Often	(Ref)	(Ref)
Sometimes/never	**2.3 (1.4 – 3.7)**	**1.9 (1.1 – 3.0)**
**House security (worried about paying rent or house loan)**		
No	(Ref)	(Ref)
Sometimes	0.8 (0.4 – 1.8)	0.8 (0.3 –1.7)
Yes	1.1 (0.6 – 2.0)	1.6 (0.9 – 2.7)
**Worried about getting evicted from house**		
No	(Ref)	(Ref)
Sometimes	**3.0 (1.5 – 6.2)**	**4.5 (2.2 – 9.3)**
Yes	**3.8 (2.0 –7.0)**	**4.5 (2.4 – 8.4)**

*Estimated using logistic regression analyses separately for the outcomes (depression and anxiety) after adjusting for city, sex, age, education, employment status, financial status, food security and house security during the lockdown. The bold values are significant values.

For participants who were only ‘sometimes worried about getting evicted from the house’, the risk of depression was 3 times and anxiety was 4.5 times more than those who were not worried (OR 3, 95% CI 1.5 – 6.2 and OR 4.5,95% CI 2.2 – 9.3). Furthermore, people who were regularly worried about getting evicted had higher odds of depression (3.8 times) and anxiety (4.5 times) than those who did not worry about evictions (OR 3.8, 95%CI 2.0 –7.0 and OR 4.5, 95% CI 2.4 – 8.4, respectively).

## Discussion

The COVID-19 pandemic has caused unprecedented changes around the globe in a very short time, affecting all the facets of people’s lives. This study assessed the levels of psychological distress measured in terms of depression and anxiety in tobacco users and who had recently quit tobacco during the COVID-19 pandemic in India. We found that, 20.5% of study participants had symptoms of depression with the majority experiencing mild depression, and 3.9% experienced moderate to severe depression. Similarly, anxiety was present in 20.7% of tobacco users, with 3.7% experiencing moderate to severe symptoms. The prevalence of anxiety and depression was lower in our study as compared to the other studies conducted among the general population in India and globally during the COVID-19 times (27–29). This could be because our study was limited to tobacco users and people who had recently quit tobacco in two large metropolitan cities of India and was conducted at much later stage of the COVID-19, when gradual easing of lockdown was in process. This was the period when there was relaxation in restrictions which might have led to a relative sense of normalcy. We did not find any association between tobacco use and anxiety and depression symptoms, which could be attributed to the fact that there was limited illegal availability of tobacco products during the ban (11).

The findings of our study showed an association between depression and anxiety symptoms (score ≥ 5) with the present unemployment, financial, food and housing insecurity. Similar findings were observed in an online study conducted in India during the pandemic among the general population where the financial status of the family and ability to access essential supplies were seen to be linked with anxiety and depression (30).

When the depression and anxiety scores were analyzed along gender lines, women had significantly higher scores than males. These findings although cannot be generalized because female population in our sample was skewed.

India’s mental health care system, which is a part of the general health care system, has suffered from sub-optimal investment and was already over-extended and under-resourced even before the advent of the pandemic. It is mostly curative in nature concentrating on providing tertiary care. COVID-19 has caused widespread social and economic turmoil across the globe. Although the Government of India has taken several initiatives to protect the most vulnerable population, there are definite gaps in its reach, nature as well level of protection it offers. Financial insecurities take a definite toll on the mental health of individuals as seen in our study. The pandemic has accentuated the need for a comprehensive social security net now more than ever. This crisis should be seen as an opportunity to rebuild a strong resilient health system to broaden the canopy of universal health coverage. Easy access to tobacco cessation services must be provided as it is crucial for both the physical and mental health of tobacco users.

### Strengths and limitations of the study

The strength of our study lies in the fact that it tries to explore the effect of life altering situations during the COVID-19 pandemic on mental health among tobacco users in India. Our questionnaire was designed based on previously validated STOP survey (31). There is a dearth of literature on mental health among tobacco users in the country. Possible limitations of this study include use of telephonic interviews for data collection. This did not allow building up of rapport with the participants which is especially crucial in sensitive topics like mental health. This may not have characterized mental health status of the people with accuracy of structured face to face interviews. There is also the possibility of recall bias because participants may not accurately recall having depression and anxiety symptoms in preceding two weeks. There is also a possibility of depression and anxiety among the participants due to some other reasons not captured through our survey. There are predictive limitations because the study is cross-sectional so causal inferences cannot be drawn. We did not have pre-COVID data on depression and anxiety levels of the study participants to compare. We did not have control group of non-tobacco users so could not assess if tobacco use was one of the correlates of mental health status. The study was conducted across two cities of India namely, Delhi and Chennai and hence, the study findings may not be generalized to entire Indian population. We tried to encompass equal number of males and females, due to societal taboo or other reason a smaller number of females agreed to be part of our study. Hence, it does not generalize our results. However, we able to study the objective of our study despite the limitations. We were not able to use a specific definition of ‘serious illness’ to exclude participants due to the multiplicity of such serious health conditions, however, these excluded participants were deemed to be ill enough so as not to be able to respond to the telephonic survey.

## Conclusion

Just over 20% of tobacco users in our study had symptoms of depression or anxiety. We did not find any association between types/number of tobacco products used and depression/anxiety. Financial, food and housing insecurity among tobacco users was associated with higher depression and anxiety levels. The measures enforced by the Government of India to reduce access to tobacco products during the nationwide COVID-19 lockdown may have led to the creation of an enabling environment for existing tobacco users to limit their tobacco use through reduced access and expenditure on these products. There is also an urgent need to prioritize universal health coverage, expanded social security net, tobacco cessation and mental health services as we increasingly face such emergency lockdown situations.

## Data availability statement

The original contributions presented in this study are included in the article/Supplementary material, further inquiries can be directed to the corresponding author.

## Ethics statement

The studies involving human participants were reviewed and approved by the Centre for Chronic Disease Control’s Institutional Ethics Committee (Reference # CCDC_IEC_04_2018). Informed consent was sought from eligible participants. A verbal consent was audio-recorded following the Indian Council of Medical Research’s revised guidelines for obtaining consent for biomedical and health research during the COVID-19 pandemic. Participants who were suffering from any severe illness, institutionalized, unable to respond the survey, and not willing to provide or record verbal consent were excluded from the study. All data was collected in accordance with guidelines, protocols and methods approved by the CCDC’s Ethics Committee. All the necessary measures to safeguard participants’ anonymity and confidentiality of information were respected. The patients/participants provided their written informed consent to participate in this study.

## Author contributions

MA, GN, and SB conceptualized the study. SB and GN led the data collection efforts and contributed to study administration. SB, GN, NJ, NS, and AP contributed to data management, analysis, interpretation of results, and drafting the manuscript. MD, SM, SP, MKA, AM, NT, DP, and MA provided technical inputs on data analysis, interpretation of results, and reviewed the manuscript critically for intellectual contents. All authors approved the final version of the manuscript and are accountable for the accuracy and integrity of any part of the work.

## Abbreviations

PHQ 9: Patient Health Questionnaire-9
GAD 7: Generalized Anxiety Disorder-7
SD: Standard deviation
CI: Confidence interval
OR: Odds ratio.
