# Supplementary material for: Anxiety and depression among adult tobacco users during the COVID-19 restrictions in India
Source: Front Psychiatry. 2022 Aug 23;13:964949. doi: 10.3389/fpsyt.2022.964949 (PMC9445265; doi:10.3389/fpsyt.2022.964949)
Supplement: Supplementary file 1 [file Data_Sheet_1.docx]

**Table S1: Disposition table**

| **Disposition Category** | **Number of Calls** |
| --- | --- |
| Total Completed Surveys (C) | 801 |
| Refusals | 138 |
| **Total eligible (E)** | 939 |
| Non-Tobacco Users | 253 |
| Wrong numbers/out of service | 568 |
| Expired | 28 |
| **Total in-eligible (I**) | 849 |
| **Known (E+I)** | 1788 |
| **Unavailable/ non-contacted/ Unknown (U )** | 717 |

**Table S2: Response rates using different methods:**

| **OVERALL** | **Delhi** | **Chennai** | **Total** | **Total Rates** | **Delhi Rates** | **Chennai Rates** |
| --- | --- | --- | --- | --- | --- | --- |
| **Total Calls** | 1365 | 1140 | 2505 |  |  |  |
| **Refusals** | 88 | 50 | 138 | **5.5** | 6.4 | 4.4 |
| **Unavailable (NC)** | 360 | 357 | 717 | **28.6** | 26.4 | 31.3 |
| **Expired** | 9 | 19 | 28 |  |  |  |
| **Wrong no/out of service** | 359 | 209 | 568 | **22.7** | 26.3 | 18.3 |
| **Not tobacco user** | 105 | 148 | 253 | **10.1** | 7.7 | 13.0 |
| **Completed surveys ( C)** | 444 | 357 | 801 | **32.0** | 32.5 | 31.3 |
| **Total eligible (E)** | 532 | 407 | 939 |  |  |  |
| **Gross Response rate= C/E +NC** | 48.36% | | | | | |
| **AFTER REMOVING THOSE WHO COULD NOT BE CONTACTED AND NON-TOBACCO USERS.** | | | | | | |
|  | **Delhi** | **Chennai** | **Total** | **Total Rates** | **Delhi Rates** | **Chennai Rates** |
| **Total eligible (E)** | 532 | 407 | 939 |  |  |  |
| **Refusals** | 88 | 50 | 138 | **14.7** | 16.5 | 12.3 |
| **Unavailable** | 360 | 357 | 717 | **76.4** | 67.7 | 87.7 |
| **Expired** | 9 | 19 | 28 |  |  |  |
| **Wrong no/out of service** | 359 | 209 | 568 | **60.5** | 67.5 | 51.4 |
| **Not tobacco user** | 105 | 148 | 253 | **26.9** | 19.7 | 36.4 |
| **Completed surveys (C)** | 444 | 357 | 801 | **85.3** | 83.5 | 87.7 |
| **Ineligible Participants** | 473 | 376 | 849 |  |  |  |
| **Basic response Rate= C/E** | 85.30% | | | | | |
| **CASRO Estimator** | **Delhi** | **Chennai** | **Total** | **Total Rates** | **Delhi Rates** | **Chennai Rates** |
| **Total eligible ( E )** | 532 | 407 | 939 |  |  |  |
| **Refusals** | 88 | 50 | 138 | **14.7** | 16.5 | 12.3 |
| **Unavailable (NC)** | 360 | 357 | 717 | **76.4** | 67.7 | 87.7 |
| **Expired** | 9 | 19 | 28 |  |  |  |
| **Wrong no/out of service** | 359 | 209 | 568 | **60.5** | 67.5 | 51.4 |
| **Not tobacco user** | 105 | 148 | 253 | **26.9** | 19.7 | 36.4 |
| **Completed surveys (C)** | 444 | 357 | 801 | **85.3** | 83.5 | 87.7 |
| **Ineligible Participants** | 473 | 376 | 849 |  |  |  |
| **Known (K)** | 1005 | 783 | 1788 |  |  |  |
| **Unknown (U)** | 360 | 357 | 717 |  |  |  |
| **CASRO Estimate= C/[E(1+(U/K))]** | 60.88% | | | | | |

**Table S3: Variable Information**

| **Outcome variables** | |
| --- | --- |
| **Depression** | The validated Patient Health Questionnaire-9 (PHQ-9) was used in our study to evaluate the severity of depressive symptoms among tobacco users. Participants rated each item-  1 )Little interest or pleasure in doing things day  2) Feeling down, depressed, or hopeless?  3) Trouble falling or staying asleep, or sleeping too much? 4) Feeling tired or having little energy?  5) Poor appetite or overeating?  6) Feeling bad about yourself or that you are a failure or have let yourself or your family down?  7) Trouble concentrating on things, such as reading the newspaper or watching television?  8) Moving or speaking so slowly that other people could have noticed? Or the opposite – being so fidgety or restless that you have been moving around a lot more than usual?  9) Thoughts that you would be better off dead, or of hurting yourself in some way?  By the incidence of symptoms over the past two weeks on a 4- point scale from 0 (not at all), 1 (several days), 2 (more than half the days) and 3 (nearly every day). The total score ranged from zero to 27, with a higher score indicating severe depression symptoms |
| **Anxiety** | The validated Generalized Anxiety Disorder-7 (GAD-7) was used in our study to evaluate the severity of depressive symptoms among tobacco users. Participants rated each item-  1) Feeling nervous, anxious or on edge?  2) Not being able to stop or control worrying?  3) Worrying too much about different think day  4) Trouble relaxing?  5) Being so restless that it is hard to sit still?  6) Becoming easily annoyed or irritable?  7) Feeling afraid as if something awful might happen?  By the incidence of symptoms over the past two weeks on a 4- point scale from 0 (not at all), 1 (several days), 2 (more than half the days) and 3 (nearly every day).  The total score ranged from zero to 21, with a higher score indicating severe anxiety symptoms |
| **Independent variables** | |
| **City** | City of residence of the participants- *Delhi* or *Chennai* |
| **Gender** | Biologic gender of the person- *male, female* or *other* |
| **Age** | Age of the person. Age was further categorized into categories |
| **Education status** | Education of the person- *illiterate, post graduate, graduate, secondary school, high school, primary*. |
| **Current Employment status**  **of the earner (Main)** | Is the earner (main) in your household currently *employed/running a business?- unemployed, employed/running a business but not currently working, employed/ running a business but currently working, uncertain* |
| **Current financial status** | How well would you say you (and your family) are managing financially right now? Would you say you are: *Doing alright, just about getting by, finding it quite difficult, don’t know, do not wish to answer* |
| **Lock down status** | Is your residential area currently in a situation of lockdown? *Yes, a containment zone, yes a red zone, yes a buffer zone, yes an orange zone, no a green zone, don’t know* |
| **Food Security (ability to buy food during COVID-19 lockdown)** | I have been able to buy the food I needed - For the given statement please tell whether the statement was *often true, sometimes true or never true* |
| **House security** | 1) Worried about paying rent or house loan- How much do you agree/disagree with the following statement today: I worry about paying house rent/house loan- *No, sometimes, yes*  2) I worry about getting evicted/losing my home- *No, sometimes, yes* |
|  |  |
| **Current cigarette use** | Participants were asked the question-  “Which of the following statements (about cigarette smoking) apply to you best now? “   \| *i) I smoke cigarettes every day* \| \| \| --- \| --- \| \|  \| *ii) I smoke cigarettes, but not every day.* \| \|  \| *iii)I smoke cigarettes, and I also smoke tobacco in other forms (e.g. bidi/water pipe/ cigar)*  *iv)I smoke cigarettes, and I also use smokeless tobacco (e.g. gutka/khaini/zarda/pan with tobacco etc.)* \| \|  \| Participants responding, yes to one of the above categories were categorized as“Yes”.  Others who responded- yes to following options were categorized as “No”. \|   *vi)I have stopped smoking cigarettes but I continue to smoke other smoked forms of tobacco (bidi/water pipe/cigar)*  *vii) I have stopped smoking cigarettes but I continue to use other smokeless forms of tobacco (e.g. gutka/khaini/zarda/pan with tobacco etc.)*  *viii) I have stopped smoking cigarettes but I continue to use electronic cigarettes*  *ix) I have stopped smoking cigarettes but now I smoke other forms of tobacco (bidi/water pipe/cigar)*  *x) I have stopped smoking cigarettes but now I use other smokeless forms of tobacco (e.g. gutka/khaini/zarda/pan with tobacco etc.)*  *xi) I have stopped smoking cigarettes but now I use electronic cigarettes*  *xii) I have completely stopped smoking cigarettes now*  . xiii) I have never smoked cigarettes. |
| **Bidi smokers** | Participants were asked the question-  “Which of the following statements (about bidi smoking) apply to you best now? “   \| *i)I smoke bidis every day* \| \| \| --- \| --- \| \|  \| *ii) I smoke bidis, but not every day.* \| \|  \| *iii)I smoke bidis, and I also smoke tobacco in other forms (e.g. water pipe/ cigar)*  *iv)I smoke bidis , and I also use smokeless tobacco (e.g. gutka/khaini/zarda/pan with tobacco etc.)* \| \|  \| Participants responding, Yes to one of above statements were categorized as “Yes”.  *v) I have stopped smoking bidis but I continue to smoke other smoked forms of tobacco (cigarettes/waterpipe/cigar)*  *vi) I have stopped smoking bidis but I continue to use other smokeless forms of tobacco (e.g. gutka/khaini/zarda/pan with tobacco etc.)*  *vii) I have stopped smoking bidis but I continue to use electronic cigarettes*  *viii) I have stopped smoking bidis but now I smoke other forms of tobacco (cigarettes/water pipe/cigar)*  *ix) I have stopped smoking bidis but now I use other smokeless forms of tobacco (e.g. gutka/khaini/zarda/pan with tobacco etc.)*  *x) I have stopped smoking bidis but now I use electronic cigarettes*  *xi) I have completely stopped smoking bidis now.*  *xii) I have never smoked bidis*  Participants who responded yes to one of above statements were categorized as *“ No”* \| |
| **SLT users** | Participants were asked the question-  “Which of the following statements (about smokeless tobacco) apply to you best now? “   \|  \| *i) I use smokeless tobacco (gutka, khaini, zarda, pan with tobacco etc.) everyday.*  *ii) I use smokeless tobacco, but not everyday.*  *iii) I use smokeless tobacco, and I also smoke tobacco (e.g. bidi, cigarette, water pipe or cigar).*  *iv) I use smokeless tobacco and I also use electronic cigarettes.*  Participants responding, Yes to one of above statements were categorized as“Yes”.  *v) I have stopped using smokeless tobacco but I continue to use electronic cigarettes*  *vi) I have stopped using smokeless tobacco but now I smoke tobacco (bidi/cigarettes/water pipe/cigar)*  *vii) I have stopped using smokeless tobacco but now I use electronic cigarettes*  *vii) I have completely stopped using smokeless tobacco now.*  *ix) I never used smokeless tobacco.*  Participants who responded yes to one of above statements were categorized as *“ No”* \| \| --- \| --- \| \|  \|  \| |
